# The Impact of Resampling and Denoising Deep Learning Algorithms on Radiomics in Brain Metastases MRI

**DOI:** 10.3390/cancers14010036

**Published:** 2021-12-22

**Authors:** Ilyass Moummad, Cyril Jaudet, Alexis Lechervy, Samuel Valable, Charlotte Raboutet, Zamila Soilihi, Juliette Thariat, Nadia Falzone, Joëlle Lacroix, Alain Batalla, Aurélien Corroyer-Dulmont

**Affiliations:** 1 Medical Physics Department, CLCC François Baclesse, 14000 Caen, France; mr.ilyassmoummad@gmail.com (I.M.); c.jaudet@baclesse.unicancer.fr (C.J.); 21509710@etu.unicaen.fr (Z.S.); abatalla@baclesse.unicancer.fr (A.B.); 2 UMR GREYC, Normandie University, UNICAEN, ENSICAEN, CNRS, 14000 Caen, France; alexis.lechervy@unicaen.fr; 3 ISTCT/CERVOxy Group, Normandie University, UNICAEN, CEA, CNRS, 14000 Caen, France; samuel.valable@cnrs.fr; 4 Radiology Department, CLCC François Baclesse, 14000 Caen, France; c.raboutet@baclesse.unicancer.fr (C.R.); j.lacroix@baclesse.unicancer.fr (J.L.); 5 Radiotherapy Department, CLCC François Baclesse, 14000 Caen, France; jthariat@gmail.com; 6 GenesisCare Theranostics, Building 1 & 11, The Mill, 41-43 Bourke Road, Alexandria, NSW 2015, Australia; falzonepnc@gmail.com

**Keywords:** deep learning, radiomics, MRI, resampling, denoising

## Abstract

**Simple Summary:**

Due to the central role of magnetic resonance Imaging (MRI) in the management of patients with cancer, waiting lists exceed clinically relevant delays. For this reason, many research groups and MRI manufacturers develop algorithms as resampling and denoising models to allow faster acquisition time without deterioration in image quality. Whereas these algorithms are available in all new MRI, it is not clear how they will impact image features as well as the validity of statistical model of radiomics which use deep images characteristics to predict treatment outcome. The aim of this study was to develop resampling and denoising deep learning (DL) models and evaluate their impact on radiomics from post-Gd-T1w-MRI brain images with brain metastases. We show that resampling and denoising DL models reconstruct low resolution and noised MRI images acquired quickly into high quality images. While fast acquisition loses most of the radiomic-features and invalidates predictive radiomic models, DL models restore these parameters.

**Abstract:**

Background: Magnetic resonance imaging (MRI) is predominant in the therapeutic management of cancer patients, unfortunately, patients have to wait a long time to get an appointment for examination. Therefore, new MRI devices include deep-learning (DL) solutions to save acquisition time. However, the impact of these algorithms on intensity and texture parameters has been poorly studied. The aim of this study was to evaluate the impact of resampling and denoising DL models on radiomics. Methods: Resampling and denoising DL model was developed on 14,243 T1 brain images from 1.5T-MRI. Radiomics were extracted from 40 brain metastases from 11 patients (2049 images). A total of 104 texture features of DL images were compared to original images with paired *t*-test, Pearson correlation and concordance-correlation-coefficient (CCC). Results: When two times shorter image acquisition shows strong disparities with the originals concerning the radiomics, with significant differences and loss of correlation of 79.81% and 48.08%, respectively. Interestingly, DL models restore textures with 46.15% of unstable parameters and 25.96% of low CCC and without difference for the first-order intensity parameters. Conclusions: Resampling and denoising DL models reconstruct low resolution and noised MRI images acquired quickly into high quality images. While fast MRI acquisition loses most of the radiomic features, DL models restore these parameters.

## 1. Introduction

Multimodal imaging is a central diagnostic tool in medicine, especially for the management of patients with cancers. From 2009 to 2019 the number of imaging examinations in the USA increased by 18% and 42% for computed tomography (CT) and magnetic resonance imaging (MRI), respectively [1]. In cancer treatment centres, MRI is used for the diagnosis and treatment follow-up of patients, which has placed a significant demand on resources. All of these factors have led to an increased delay in obtaining an MRI appointment with waiting times now up to weeks or month(s) in France/Europe (30 days on average) [2]. There is thus a pressing unmet need to reduce MRI acquisition time, to allow for better patient management. To reduce acquisition time, several approaches have been developed, such as partial Fourier transforms and parallel imaging. However, these techniques cause significant image degradation [3,4]. Compressed sensing, a signal processing technique for efficient signal acquisition and reconstruction by finding solutions to underdetermined linear systems, undersample the k-space. This allows for shorter acquisition times and estimation of the non-acquired k-space data through an iterative process [5]. This technique is frequently used in clinical settings to reduce acquisition times but has reached its limits and delays to obtain an MRI acquisition remain too long.

Artificial intelligence (AI) and especially deep learning (DL) -a subset of AI- has seen massive development in medical systems in the last five years [6]. Beyond the use of DL for automated diagnosis from radiology images [7], DL can be used to accelerate image acquisition. In 2019, the New York University Langone Health and Facebook AI Research consortium created a fast MRI challenge [8] to investigate the use of DL to make MRI scans faster while maintaining high image quality. The dataset released contained k-space data, and the idea was to simulate acquisition matrix subsampling by applying a mask in the Fourier domain and learn the mapping between subsampled images and fully sampled images. Thanks to the advances of DL algorithms, manufacturers now sell MRI machines equipped with DL algorithms to accelerate scan times combining them for example with compressed sensing algorithms [9] or with DL denoising algorithms that work directly on the final image, reducing the number of measurements of the signal or number of excitation (NEX) [10]. Finally, in this constantly progressive research field, numerous algorithms have been developed. The most adapted DL architecture model for medical image restoration is convolutional neural networks (CNN) [11] with efficient sub-pixel convolutional neural network (ESPCN) [12] or fully convolutional network (U-Net) [13] architecture.

Despite the Food and Drug Administration (FDA) and European Medicines Agency agreement for routine clinical use, it is not clear how these algorithms will impact the image or affect the validity of statistical models derived from radiomics. Radiomics provide a methodology to extract different features based on intensity, shape or texture from images in order to build predictive models [14]. This approach holds great promise to predict patient prognosis, treatment response or the identification of molecular markers. For example, an overall survival predictive model including radiomics features was computed in lung cancer [15]. A recent review by Lohmann et al. studied the models in patients with brain metastases [16]. They presented 13 radiomic models with an area under the curve (obtained with a receiver operating characteristic curve and which define the specificity and the sensibility) between 0.73 and 0.98 to differentiate between glioblastoma (GBM) and BM, prediction of BM origin, treatment response and overall survival.

This very promising emerging field has numerous pitfalls that have been identified by the radiomics community including study design, data acquisition, segmentation, features calculation and modelling [17]. The stability of these predictive models has to be challenged before their adoption as a standard of care. In this study, we focus on the effect of AI denoising and resampling on radiomics predictive models.

A study of radiomic feature reproducibility with DL algorithms is mandatory in the new generation MRI machines using DL algorithms to confirm the validity of radiomic analysis. We propose using DL models for resampling and denoising to accelerate acquisition time by a factor of ~2 (dividing by two the acquisition matrix and the NEX respectively), and to investigate the impact of these DL models on radiomic feature reproducibility.

## 2. Materials and Methods

### 2.1. Patients

This retrospective study was approved by the local institutional review board. Eighty-five patients presenting with brain metastases (BM) referred to our oncological center between January 2017 and December 2019 were included. Post-Gd T1 brain imaging was undertaken for initial diagnosis or treatment efficacy follow-up. MR-004, a national French institution (INDS) defining health research conduct guidelines was used for this study. The study population characteristics are shown in Table 1.

### 2.2. Magnetic Resonance Imaging (MRI) Acquisition

MRI was performed on an AREA SIEMENS 1.5 Tesla magnet using a brain dedicated 16 channels coil with the patient in a supine position. Prior to the examination patients were injected with 0.2 mL/kg of DOTAREM (500 µmol/mL). After a shimming process and scout imaging scan, tumor gadolinium enhancement was detected with a post-Gd T1 brain sequence (TR/TEeff = 2070/3.15 ms; Angle = 15°; NEX = 1; 208 contiguous slices; resolution = 0.5 × 0.5 × 1 mm; acquisition matrix = 256 × 256 pixels and acquisition time = 4 min 48).

### 2.3. Dataset, Resampling and Denoisning DL Models

To reduce the acquisition time in MRI, acquisitions can be made with half the acquisition matrix or half the NEX. However, this leads to undersampled and noisy images. Therefore, a supervised learning approach was used to “learn” a function that maps low quality images (acquired rapidly) to high quality images (acquired slowly). As it was not possible to obtain true downsampled and noisy images owing to the limited availability of the MRI, downsampled and noisy images were simulated by halving the acquisition matrix using linear interpolation to decrease spatial resolution and adding Rician noise in the MRI image, respectively. The NEX was halved by transforming the image in the Fourier domain then adding an additive random Gaussian noise both to the real part and the imaginary part. The magnitude of the noisy complex image was then computed before finally transforming it back to the spatial (pixel) domain [18,19]. The flow diagram of the method used in this study is presented in Figure S1.

**Figure 1:**
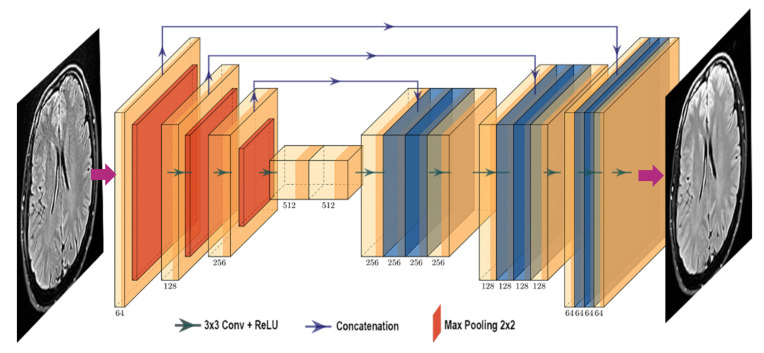
Deep learning model architecture.

DL models were developed with a total of 14,243 unique post-Gd T1 brain images obtained from 85 acquisitions which were split into 9756, 2438 and 2049 images for training, validation, and testing, respectively. DL models were developed using the Keras python library [20] which is based on a U-Net architecture [21], described in Figure 1.

The loss function used to train the model is described below:

(1) $$MixE\left(Y,\;\hat{Y}\right)=MSE\left(Y,\;\hat{Y}\right)+0,\;1\;MGE\left(G_{Y},{\hat{G}}_{\hat{Y}}\right)+0,\;1\;MS\_SSIM\left(Y,\;\hat{Y}\right)$$

With:

(2) $$MSE\left(Y,\;\hat{Y}\right)=\frac{1}{mn}\;\overset{m}{\underset{i=1}{\sum }}\overset{n}{\underset{j=1}{\sum }}{\left(\hat{Y}\left(i,j\right)-Y(i,j)\right)}^{2}$$

(3) $$MGE\left(G_{Y},{\hat{G}}_{\hat{Y}}\right)=\frac{1}{mn}\;\overset{m}{\underset{i=1}{\sum }}\overset{n}{\underset{j=1}{\sum }}{\left({\hat{G}}_{\hat{Y}}\left(i,j\right)-G_{Y}(i,j)\right)}^{2}$$

(4) $$G_{Y}\left(i,j\right)=\sqrt{G_{Y}{}_{x}^{2}\left(i,j\right)+G_{Y}{}_{y}^{2}\left(i,j\right)}$$

(5) $$G_{Y}{}_{x}=Y*[\begin{array}{ccc} -1 & -2 & -1\\ 0 & 0 & 0\\ 1 & 2 & 1 \end{array}]$$

(6) $$G_{Y}{}_{y}=Y*[\begin{array}{ccc} -1 & 0 & 1\\ -2 & 0 & 2\\ -1 & 0 & 1 \end{array}]$$

(7) $$SSIM\left(P_{\hat{Y}},P_{Y}\right)=\frac{1}{N}\underset{P_{\hat{Y}}\;P_{Y}}{\sum }\frac{\left(2\mu_{P_{\hat{Y}}}\mu_{P_{Y}}+c_{1}\right)\left(2\sigma_{P_{\hat{Y}}}\sigma_{P_{Y}}+c_{2}\right)}{\left(\mu^{2}{}_{P_{\hat{Y}}}+\mu^{2}{}_{P_{Y}}+c_{1}\right)\left(\sigma^{2}{}_{P_{\hat{Y}}}+\sigma^{2}{}_{P_{Y}}+c_{2}\right)}$$

where:

*MSE*: Mean Squared Error
*MGE*: Mean Gradient Error
*MS_SSIM*: Multi-Scale SSIM corresponding to multiple SSIM image evaluations at different image scales [22].
*N*: Number of batch over which SSIM has been averaged
$\mu_{P_{Y}}$ and $\mu_{P_{\hat{Y}}}$: Mean of patches *P_Y_* and $P_{\hat{Y}}$, respectively
$\sigma_{P_{Y}}$ and $\sigma_{P_{\hat{Y}}}$: Deviation of patches *P_Y_* and $P_{\hat{Y}}$, respectively
$c_{1}$ and $c_{2}$: Constants

The Python code for the resampling and denoising DL model is available at: https://github.com/AurelienCD/Resampling_Denoising_Deep_Learning_MRI (accessed on 21 December 2021).

The quality of the model was then evaluated comparing the peak signal-to-noise ratio (PSNR, formula (8)) and the structural similarity method between input and output images with the original MRI image as reference. The input and output imaging format was DICOM.

(8) $$PSNR\left(Y,\;\hat{Y}\right)=10\;log_{10}\left(\frac{MAX_{I}^{2}}{MSE\left(Y,\;\hat{Y}\right)}\right)\;$$

$MAX_{I}$ is the maximum possible pixel value of the image.

### 2.4. Image Processing, Radiomics Extraction and Analysis

All image processing was performed using a 3D slicer version 4.10 [23] and ImageJ software [24]. A total of 40 BM contours from 11 patients were obtained using the 3D slicer segmentation program designed for brain tumors [25]. Volumes of interest (VOIs) obtained from the original images were also used on DL input and output images.

Radiomic feature values were extracted from BM VOI using the Pyradiomics python library [26] as previously described [27]. Through radiomics extraction in accordance with the Imaging Biomarker Standardization Initiative (IBSI) [28], up to seven classes of features can be obtained. (1) The First-order intensity class describes the distribution of pixel values. (2) The Gray Level Co-occurrence Matrix (GLCM) class describes the occurrence of similar pixel values in the image. (3) The Gray Level Size Zone Matrix (GLSZM) features quantify gray level zones in an image. A gray level zone is defined as the number of connected voxels that share the same gray level intensity in three dimensions. (4) The Gray Level Run Length Matrix (GLRLM) class evaluates the gray level runs, which are defined as the length in number of pixels, of consecutive pixels that have the same gray level value in one dimension. (5) The Neighboring Gray Tone Difference Matrix (NGTDM) describes the difference between a gray value of a pixel and the average gray value of neighbors. (6) The Gray Level Dependence Matrix (GLDM) characterizes the number of connected voxels within a distance from the center voxel in function of their grey level. (7) The IQ wavelets class contains two features, a local analysis of the VOI only and a global analysis of the whole image. These metrics characterize image quality as the ratio between high and low wavelet frequencies. To evaluate the impact of DL algorithms on radiomics, we compared predictive radiomic models values before and after DL algorithm processing. Two predictive models of radiomic were used base on [29,30], which are radiomic models for the prediction of treatment response (overall survival) of BM from NSCLC and BM classification. More details on the radiomic models can be found in Table S2.

Lastly, to evaluate the performance of the DL model in comparison with the twice-shorter acquired images (downsampled or noisy images), maps of change in pixel value between post-processing and reference images were computed with ImageJ as follows:

$$\frac{\mathrm{abs}\left(\mathrm{Postprocessing}\;\mathrm{image}-\mathrm{reference}\;\mathrm{image}\right)}{\mathrm{reference}\;\mathrm{image}}*100$$

### 2.5. Statistical Analysis

Data are presented using boxplots with minimum, maximum, 1st quartile and 3rd quartile. *p*-values < 0.05 were considered statistically significant. A paired student’s *t*-test was used to compare features in original and DL images. The correlation between original, fast and DL images was analysed with a Pearson test and the Concordance Correlation Coefficient (CCC) [31]. CCC values of ± 1 describe a perfect positive/negative correlation respectively and a value of 0, no correlation. Features with a minimum CCC of 0.85 were considered as statistically reproducible and concordant and the radiomic values stable [32]. All the statistical analysis were performed using python [33] and SciPy library. All python codes used in the analysis are available on https://github.com/AurelienCD/Resampling_Denoising_Deep_Learning_MRI (accessed on 21 December 2021). Finally, to more deeply understand the mechanism of the DL models, scatter plots of radiomics features for original and DL images were performed.

## 3. Results

### 3.1. Resampling DL Model

#### 3.1.1. Quality of the Resampling DL Model

**Figure 2:**
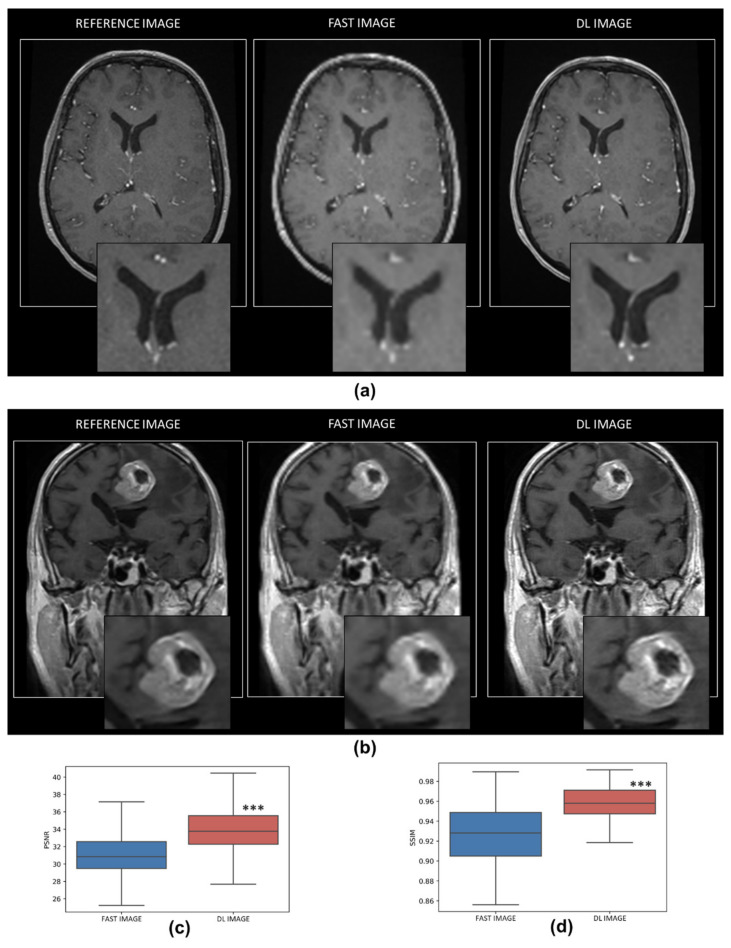
Resampling DL model. Representative magnetic resonance imaging (MRI) of reference images, fast acquisition images and DL reconstruction images in healthy (**a**) and pathological conditions (**b**). Quantitative analysis of the efficiency of the resampling DL model with the comparison with fast acquired image concerning PSNR (**c**) and SSIM (**d**) metrics. *n* = 2049 for both groups, *** *p* < 0.001 vs. fast acquired image.

As expected, and presented in Figure 2, fast image with an acquisition matrix divided by two, present with low resolution with or without an underlying pathologic condition. More interestingly, the DL resampling model was able to reconstruct well-defined MRI images with an increase in PSNR and SSIM values in comparison to fast acquired images (PSNR: 31.44 ± 2.89 vs. 34.24 ± 2.80, *p* < 0.001 and SSIM: 0.93 ± 0.03 vs. 0.96 ± 0.03, *p* < 0.001 for fast and DL images, respectively).

**Figure 3:**
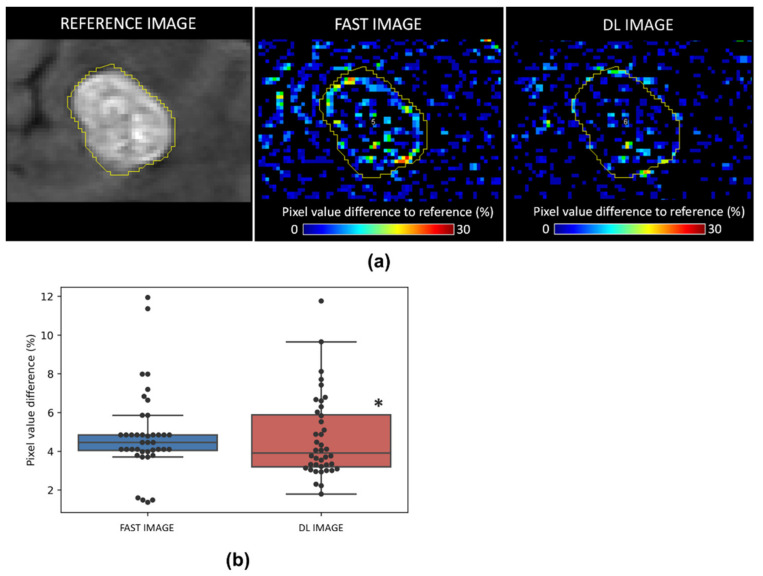
Effect of resampling DL model on BM signal intensity. (**a**) Representative MRI of reference images (left), difference map with fast acquisition images (middle) and DL reconstruction images (right). (**b**) Quantitative analysis of pixel value difference (%) in fast and DL images. *n* = 40 for both groups, * *p* < 0.05 vs. fast acquired image. Bars represent minimum and maximum values.

To investigate the impact of DL reconstruction on BM signal intensity, difference maps from the reference MRI image were computed and are presented in Figure 3. The BM signal intensity was significantly closer to the reference image in comparison to the fast image (difference value (%) 4.88 ± 2.17 vs. 4.67 ± 2.13, *p* < 0.05, for fast and DL images, respectively).

#### 3.1.2. Impact of Resampling DL Model on Radiomics Features

The stability of the radiomics features after fast acquisition and DL reconstruction was investigated in BM lesions. Paired *t*-test analysis showed that fast images present marked disparities compared to the original images with significant differences in up to 83 of the 104 texture parameters (79.81%). Particularly, there were significant differences between the basic intensity values, such as minimum, maximum, mean, median and coefficient of variation (*p* < 0.05). Interestingly, the DL reconstruction allowed a restoration of the majority 48/104 (46.15%) of the previously unstable parameters with an absence of significant difference for the basic intensity parameters previously mentioned, except the coefficient of variation as presented in Table 2.

A significant difference in values was observed; however, these values were highly correlated if the differences in values are the same for all the data. In that case, radiomic predictive models would be valid as the difference between responders and non-responders are maintained. To test this hypothesis, the correlation (CCC) between radiomic values in reference and post-processing (fast and DL reconstructed images) were analyzed.

**Figure 4:**
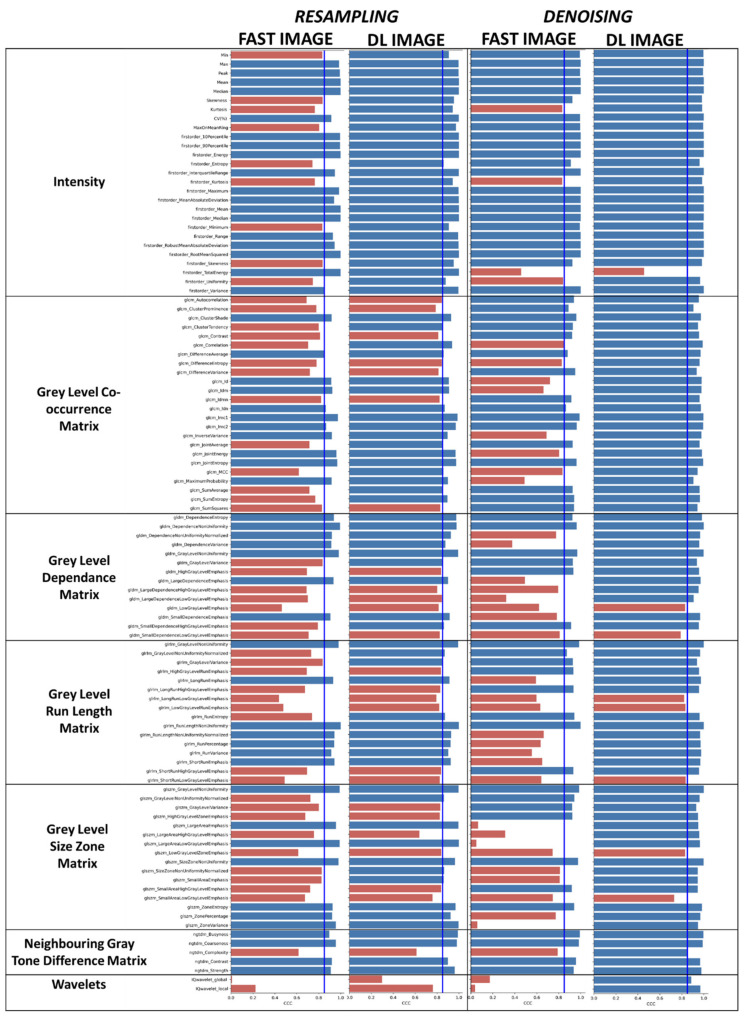
Effect of fast acquisition and resampling DL model on the correlation between reference and post-processing image radiomic values. Red bars represent unstable radiomic features below a CCC value threshold of 0.85. Blue bars represent stable radiomic features after fast acquisition or resampling DL reconstruction.

CCC values comparing fast images and DL images with the reference images were analyzed and are presented in Figure 4, left-part. The marked disparities between the fast images and the reference images in terms of radiomic values is concomitant to a loss of correlation as demonstrated by the CCC below 0.85 for 50/104 (48.08%) of the feature parameters. Interestingly, the DL model restores the correlation of the majority of the parameters as only 27/104 (25.96%) features have a CCC below 0.85 (Figure 4, left-part). It is of note that all the intensity features were stable after resampling DL reconstruction.

**Figure 5:**
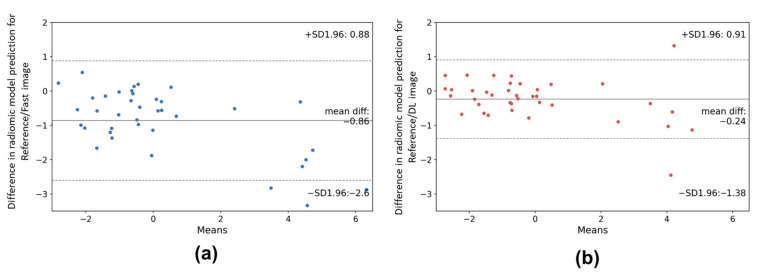
Bland–Altman plots showing the difference between predictive values obtained from the radiomic model [29] and reference images to fast downsampling images (**a**) and DL resampling image (**b**) in brain metastatic lesions.

To further explore the radiomic stability evaluation after DL processing, we evaluated the difference in predictive radiomic model results after DL resampling or fast images in comparison with reference images. Published overall survival [29] and classification [30] predictive models were used in this study. As shown in Figure 5, Bland–Altman plots highlight important differences in predictive values obtained with reference and fast images (mean difference = −0.86, *p* < 0.001). In comparison, predictive values obtained from DL images are slightly different from the values obtained from reference images (mean difference = −0.24, *p* < 0.05).

A similar approach was undertaken with another radiomic model [30] which showed significant differences in predictive values for the fast images and non-significant differences for the DL images when compared to the reference images (mean difference = −0.36 and −0.07 for fast image and DL image, respectively and *p* < 0.001 for fast image, Figure S2a,b).

### 3.2. Denoising DL Model

#### 3.2.1. Quality of the Denoising DL Model

**Figure 6:**
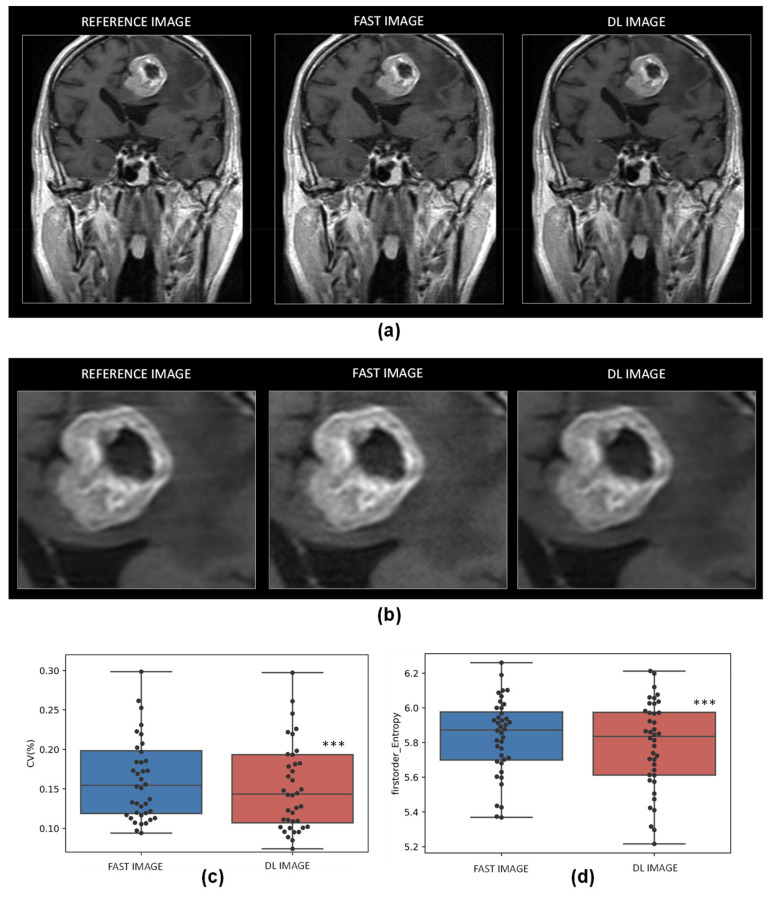
Denoising DL model. Representative MRI of reference images, fast acquisition images and DL reconstruction images in whole brain (**a**) and brain metastases (**b**). Quantitative analysis of the efficiency of the denoising DL model in brain metastases regions in comparison with fast images as evaluated by the coefficient of variation (**c**) and entropy (**d**) metrics. *n* = 40 for both groups, *** *p* < 0.001 vs. fast acquired image.

As observed in Figure 6, the denoising DL model was able to reduce noise in the fast image and produce a high quality image similar to the reference image both in the healthy and pathological tissues. For the whole image, PSNR and SSIM were significantly increased with the use of the model (PSNR: 35.48 ± 6.2 vs. 41.32 ± 4.93, *p* < 0.001 and SSIM: 0.74 ± 0.18 vs. 0.96 ± 0.04, *p* < 0.001 for fast and DL images, respectively, (Figure S3). In the brain metastases regions, the coefficient of variation (CV) and entropy, which both reflect the noise in the image, were reduced in brain metastases in the DL images (CV (%) 0.17 ± 0.08 vs. 0.16 ± 0.08, *p* < 0.001, for fast and DL images, respectively, and entropy 5.83 ± 0.22 vs. 5.79 ± 0.25, *p* < 0.001, for fast and DL images, respectively, (Figure 6).

#### 3.2.2. Impact of Denoising DL Model on Radiomics Features

The stability of the radiomics features after denoised DL reconstruction was then investigated in BM lesions. Paired *t*-test analysis showed that images reconstructed in half the time present were significantly different for 75 of the 104 texture parameters (72.12%) from the original images. The denoising DL reconstruction restored the majority of these parameters. There were 40/104 (38.46%) residual unstable parameters (Table S1). Interestingly, even if radiomics classes such as Gray Level Size Zone Matrix remained stable, significant differences compared with the reference images were observed for the intensity parameters as mean, min, max and coefficient of variation were observed (*p* < 0.01) However, as previously mentioned, radiomic features could remain stable even with a significant difference in values compared to the reference image. CCC was then evaluated and showed that, for intensity radiomic class, only first order entropy is unstable after denoising DL reconstruction (Figure 4, right part). For the other radiomic classes, only Gldm_Small Dependence Low Gray Level Emphasis and Glszm_Small Area Low Gray Level Emphasis were with a CCC < 0.8. There were 8/104 (7.69%) residual unstable radiomic features for the denoising DL reconstruction only, in comparison to the 40/104 (38.46%) unstable radiomic features for fast images acquisition. Wavelets, which are a ratio of high to low frequencies in the image, i.e., the noise in the image, were, as expected, strongly affected by fast acquisition (CCC < 0.1 and < 0.2 for local and global wavelets, respectively). It is interesting to note that, as a proof of the efficiency of the denoising DL model, wavelet features had a CCC > 0.85.

**Figure 7:**
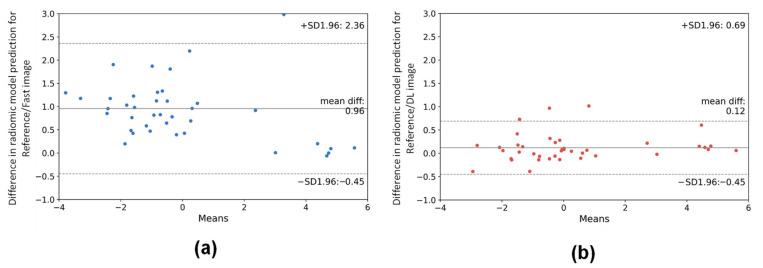
Bland–Altman plots showing the difference between predictive values obtained from radiomic model [29] from reference image to fast noising image (**a**) and DL denoising image (**b**) in brain metastases lesions, *n* = 40 for both groups.

As for the resampling DL model, a difference in the predicted radiomic model results after DL denoising in comparison to fast and reference images were evaluated. Published overall survival [29] and classification [30] predictive models were used in this study. As shown in Figure 7, Bland–Altman plots highlight important differences in predictive values obtained with reference and fast images (mean difference = 0.96, *p* < 0.001). In comparison, predicted values obtained from DL images were slightly different from the values obtained from reference images (mean difference = 0.12, *p* < 0.05).

Results for the other radiomic model [30] showed significant differences in predicted values for the fast images and non-significant differences for the DL images (mean difference = 0.15 and 0.01 for fast image and DL images, respectively. and *p* < 0.001 for fast images, Figure S2c,d).

## 4. Discussion

Long acquisition times result in unacceptable delays in patient access to MRI examinations. For this reason, many research groups, MRI manufacturers and digital startups in medical imaging are actively developing resampling and denoising models to allow faster acquisition times without a loss in image quality. Classical methods used bicubic interpolation [34] to create new neighboring pixels to upsample the image, but the resulting images were artificially smooth with some interpolation artifacts. For denoising, the state-of-the-art classical denoising method, BM3D [35], achieves good image quality without noise but smooths the image. A specified sigma value is thus required to remove noise but this may in turn remove important details in the image, critical for diagnostic images. In recent years, many deep learning architectures have been introduced for resampling (or super resolution) such as Efficient Sub-Pixel Convolutional Neural Network) (ESPCN) [12], that uses a sub-pixel convolution layer at the end to reconstruct the high resolution image [36,37] or U-NET. In our study, we initially used ESPCN architecture as a resampling solution. However, as shown in Figure S4, some cerebral structures were lost after the algorithm reconstruction. U-NET, another deep learning architecture, was recently proposed for image segmentation; however, it has shown additional utility in image resampling owing to its encoder-decoder architecture using concatenation layers to allow more information to be retained from previous layers of the network [13]. In our study, U-NET architecture was better than ESPCN for resampling purposes as it enabled the reconstruction of small cerebral structures with improved quality image metrics, such as PSNR and SSIM (*p* < 0.001), with a decrease in pixel value differences in healthy or tumour regions in comparison to reference images (*p* < 0.001, Figure S4). These differences may be due to the fact that ESPCN architecture failed to capture local information since ESPCN does not use downsampling/upsampling layers combined with skip connections for extraction of local information and because the convolution layers are applied to large size maps. However, the U-NET model used in this study could be improved by using a more complex deep learning model such as Generative Adversarial Networks (GAN). However, GAN models need large volumes of images and can elucidate some non-existing information which is a critical point for clinical imaging used for diagnosis [38]. Unsupervised models such as Deep Image Prior could also be an interesting alternative, however the reconstruction process is very long (taking a minimum of 1000 iterations ~5 min to reconstruct one image) and would not be possible for routine purposes in clinic [39].

For denoising, Denoising Convolutional Neural Network (DnCNN) is a state-of-the-art denoising method and is very efficient at removing additive white Gaussian noise [40]. However, MRI noise is not Gaussian additive and can be approximated by Gaussian noise in both the imaginary and the real parts of the k-space [18,19].

When trying to denoise MRI images, the model could confuse some important details (for example veins) for noise and remove them [41]. To avoid this problem, Gondara and colleagues [42] showed that autoencoders using convolutional layers are efficient for medical image denoising even for high noise levels, while others have shown that this model does not require a large training set to give good results [21]. For these reasons we decided to use a U-NET type architecture for both MRI resampling and denoising. We remove from vanilla (original) U-NET architecture the batch normalization as Zhang and colleagues found that they deteriorate the accuracy of image super-resolution tasks [43].

Finally, concerning the loss function, in this study we used a mix loss that combines MSE, MGE and SSIM which proved to be efficient for reconstructing low level details and structures. MSE on its own can lead to a pixel-wise average of plausible solutions which result in lack of high-frequency details (such as edges and textures) [44].

The choice of the image used for model training purposes is crucial. True fast images are hardly feasible to obtain as MRI acquisition time is already at a premium. It would require two acquisitions per patient of the same sequence adding unnecessary time to the total workflow. For this reason, all the literature publications on medical imaging simulate the noise or the downsampling from the reference images. Simulated downsampling can be obtained with bicubic interpolation [12] (with or without blurring the image using a Gaussian kernel) [45] of the reference image. In this study we choose DnCNN degradation (downsampling followed by upsampling) because it introduces degradation while preserving the image size (our model architecture requires that input and output sizes are the same) [40]. In fast MRI, they went further and undersampled the image in the Fourier domain to simulate k-space undersampling. Their approach is a better approximation to the real acquisition but it requires k-space data before any processing, which is not possible in the large majority of the clinical centers.

CNNs can be efficient at removing motion artefacts from MR images. Authors have generated motion artefacts on MR images of the liver by simulating the phase error in k-space and have trained a fully convolutional network to remove this artefact [46]. In our study, without explicitly training our denoising model to remove motion artefacts, the model captured it as if it were MRI noise and was able, in some cases, to remove it successfully (Figure S5).

The family of features were differently affected by fast imaging and DL reconstruction, (Figure 4). The intensity features that did not account for the spatial position of the voxel, which were restored from 66 to 100% by DL resampling and from 85 to 96% by DL denoising. The occurrence (GLCM) features were refurbished from 46 to 71% and from 66 to 100%, respectively. This family of features depends on the volume and quantification level but also on SNR and contrast. One-dimensional features (GLRLM) were the most affected by fast sampling from 44 to 62% and from 50 to 81% by DL resampling and DL denoising, respectively. If the spatial position of the voxel was accounted for (GLDM, GLSZM, NGTDM) the number of stability features increased from 51 to 66% for resampling and from 48 to 89% for denoising. Halving the acquisition matrix and DL resampling lead to less stable features than decreasing the number of NEX and DL denoising.

Predictive models based on radiomics is a fast-evolving field. Outstanding advances have been made since 2014 [47]. One of the main challenges still to be addressed is the interoperability and stability of these models. Over the last decade numerous studies have evaluated the impact of these bias factors in multimodal imaging [17]. Lambin and colleagues have evaluated the radiomics stability over test-retest in diffusion MRI in ovarian, colorectal and lung cancers [32] and 4DCT [48], CT [49] and PET in lung cancer [47]. These studies showed radiomic stability for 25 to 71% of all radiomic features. We observed the same order of magnitude in radiomic stability in our study for fast imaging reconstruction (>50% and >61%, respectively, for resampling and denoising) which was much lower than that obtained by DL reconstruction (>74% and >92% for resampling and denoising, respectively). The results of the previous study mentioned and the results of our study suggest that patient positioning during test-retest, magnetic field and the MRI manufacturer have a greater impact than DL reconstruction. DL algorithms are now proposed by most vendors with the release of new generation MRIs It is therefore important to evaluate the validity of these radiomic models. To the best of our knowledge, this the first study evaluating the impact of DL acceleration on radiomic stability in BM at the clinical level.

In our study we analysed the stability of the radiomic features using Pearson’s correlation and CCC. We chose to use both correlation factors as they measure different parameters. Pearson’s correlation is a measure of linearity whereas CCC is a measure of agreement. For the resampling approaches, Pearson’s correlation showed significant correlation between the fast image and DL image compared with the reference images for all the parameters. However, if we consider the CCC of the Kurtosis and Skewness features, the non-stable features with fast image reconstruction become stable after DL reconstruction. CCC thus seems a more sensitive measure with correlation clearly increased (Figure S6). Glcm-MCC, which represents the complexity of the signal in the ROI, is not stable after DL reconstruction but Person’s correlation is increased in comparison with the fast image.

We show that unstable radiomic features after resampling the DL reconstruction had a greater effect than the denoising DL reconstruction. We hypothesize that this could be due to the fact that downsampling deletes information whereas noise covers the information without deleting it. As a result, the denoising model will have to characterize the noise before its removal [50].

Finally, radiomic model validity after DL processing was evaluated as a final output. In this study we showed that whereas predictive values were strongly modified after fast acquisition, differences in predictive values after DL image processing in comparison to the reference was less important for one radiomic model [29] and with no difference for another one [30]. It is important to note that these radiomic models were obtained using a combination of radiomic features. We summarize in Table S2, the radiomic features used in the two radiomic models reported on in this study (top and middle rows of the table) and another classification model from the literature ([51], bottom row of the table). We observe that for the resampling purpose, radiomic features were unstable for 13/19 of the features used for the radiomic models, compared with only 3/19 unstable features after DL resampling processing. Concerning the radiomic models non-tested in this study, only 4/21 were unstable after DL resampling. More interestingly, the DL denoising model worked better with no unstable radiomic features for the two radiomic models [29,30] used in this study compared with only one for the radiomics model for classification used in the Qian study [51]. In regards to clinical implication, we assessed the impact of DL processing on the validity of predicted values from radiomic models. To do that, we used Bland–Altman plots and analysed the accuracy of the predicted values from radiomic model after DL processing in comparison to the original predicted values. Considering radiomic model from [29], only 2 and 3 predicted values are not accurate after resampling and denoising DL process, respectively, which represent accuracies of 95 and 92.5% (same results were obtained with radiomic model from [30]). These results highlight the impressive ability of DL to capture the shape and very precise features of the reference/high quality images during the training step and then re-inject them into new downsampled or denoised images.

As a limitation, our study was a retrospective study, as the problematic here is the delay in obtaining an MRI appointment it was not possible to negatively impact the patient’s medical path in adding for example, MRI sequences. For this reason, further prospective studies need to be undertaken for clinical validation.

Not to diminish the importance of DL reconstruction on radiomics stability, of far greater importance is the impact that MRI parameters as defined by different vendors or as used in different clinical settings can have on radiomics stability. For example, Lambin and colleagues showed that some radiomics in MRI are unstable during test-retest in multiple clinical centres [32], as well as during FDG-PET test-retest [47] or CT test-retest [49]. Knowing that, we could hypothesise that if a DL algorithm was trained using sufficient MRI images from a large number of imaging centres, it could facilitate harmonization of the image data between the different centres. In turn, this approach could standardise MRI imaging data in multicentric clinical trials similar to what the EARL approach achieved for PET imaging [52,53]. Further multicentric studies are necessary to validate this hypothesis.

## 5. Conclusions

The DL model developed in this study allows 128 × 128 pixel images with a number of average (NEX) of 1, to be reconstructed as 256 × 256 T1 images of good quality, similar to the reference image acquired in clinical routines with a NEX of 2 and an acquisition time twice as long. Concerning the texture parameters, while rapid, fast MRI acquisition loses most of the radiomic features in particular with regards to the first order intensity values. Pearson correlation and CCC analysis shows that DL models allow for the restoration of the majority of the radiomic characteristics of the original image. Finally, the majority of the radiomic features used to compute predictive radiomic models are restored after DL algorithms. This first study, which would need to be confirmed by other studies, highlights the possibility of using DL reconstructed MRI images of brain metastases for predictive radiomic model purposes.

## Figures and Tables

**Table 1:** Description of the patient cohort.

Included Patients (N)	85	Number
Sex	58	Female %
Age (Y)	66.48 ± 10.31	Mean ± SD
	(46–88)	[range]
Origin of BM		Number (%)
Lung	42 (48%)	
Breast	28 (32%)	
Kidney	6 (6.9%)	
Digestive System	3 (3.4%)	
Melanoma	3 (3.4%)	
Gynecologic	2 (2.3%)	

**Table 2:** Paired *t*-test, of DL resampling impact on radiomic features. Green highlight shows stable radiomic features values. * *p* < 0.05, ** *p* < 0.01 or *** *p* < 0.001 vs. features in the original image.

Classes	Features	Signicantly Different	Classes	Features	Signicantly Different
Intensity	Min	NS *p* = 0.73	Gray Level Dependence Matrix	gldm_DependenceEntropy	***
				gldm_DependenceNonUniformity	*
				gldm_DependenceNonUniformityNormalized	**
	Max	NS *p* = 0.47		gldm_DependenceVariance	*
	Peak	NS *p* = 0.27		gldm_GrayLevelNonUniformity	NS *p* = 0.56
	Mean	NS *p* = 0.07		gldm_GrayLevelVariance	NS *p* = 0.69
	Median	NS *p* = 0.06		gldm_HighGrayLevelEmphasis	NS *p* = 0.10
	Skewness	*		gldm_LargeDependenceEmphasis	**
	Kurtosis	NS *p* = 0.36		gldm_LargeDependenceHighGrayLevelEmphasis	**
	CV(%)	*		gldm_LargeDependenceLowGrayLevelEmphasis	NS *p* = 0.81
	MaxOnMeanRing	NS *p* = 0.08		gldm_LowGrayLevelEmphasis	NS *p* = 0.59
	firstorder_10Percentile	NS *p* = 0.14		gldm_SmallDependenceEmphasis	**
	firstorder_90Percentile	**		gldm_SmallDependenceHighGrayLevelEmphasis	NS *p* = 1.00
	firstorder_Energy	NS *p* = 0.16		gldm_SmallDependenceLowGrayLevelEmphasis	NS *p* = 0.55
	firstorder_Entropy	NS *p* = 0.97	Gray Level Run Length Matrix	glrlm_GrayLevelNonUniformity	NS *p* = 0.87
	firstorder_InterquartileRange	NS *p* = 0.10		glrlm_GrayLevelNonUniformityNormalized	NS *p* = 0.83
	firstorder_Kurtosis	NS *p* = 0.36		glrlm_GrayLevelVariance	NS *p* = 0.71
	firstorder_Maximum	NS *p* = 0.47		glrlm_HighGrayLevelRunEmphasis	NS *p* = 0.10
	firstorder_MeanAbsoluteDeviation	NS *p* = 0.13		glrlm_LongRunEmphasis	**
	firstorder_Mean	NS *p* = 0.07		glrlm_LongRunHighGrayLevelEmphasis	*
	firstorder_Median	NS *p* = 0.10		glrlm_LongRunLowGrayLevelEmphasis	NS *p* = 0.64
	firstorder_Minimum	NS *p* = 0.73		glrlm_LowGrayLevelRunEmphasis	NS *p* = 0.61
	firstorder_Range	NS *p* = 0.46		glrlm_RunEntropy	NS *p* = 0.52
	firstorder_RobustMeanAbsoluteDeviation	NS *p* = 0.20		glrlm_RunLengthNonUniformity	*
	firstorder_RootMeanSquared	NS *p* = 0.05		glrlm_RunLengthNonUniformityNormalized	**
	firstorder_Skewness	*		glrlm_RunPercentage	**
	firstorder_TotalEnergy	NS *p* = 0.15		glrlm_RunVariance	**
	firstorder_Uniformity	NS *p* = 0.89		glrlm_ShortRunEmphasis	**
	firstorder_Variance	NS *p* = 0.11		glrlm_ShortRunHighGrayLevelEmphasis	NS *p* = 0.11
Gray Level Co-occurrence Matrix	glcm_Autocorrelation	*		glrlm_ShortRunLowGrayLevelEmphasis	NS *p* = 0.63
	glcm_ClusterProminence	NS *p* = 0.81	Gray Level Size Zone Matrix	glszm_GrayLevelNonUniformity	**
	glcm_ClusterShade	NS *p* = 0.81		glszm_GrayLevelNonUniformityNormalized	**
	glcm_ClusterTendency	NS *p* = 0.86		glszm_GrayLevelVariance	NS *p* = 0.90
	glcm_Contrast	**		glszm_HighGrayLevelZoneEmphasis	NS *p* = 0.88
	glcm_Correlation	***		glszm_LargeAreaEmphasis	NS *p* = 0.16
	glcm_DifferenceAverage	**		glszm_LargeAreaHighGrayLevelEmphasis	NS *p* = 0.07
	glcm_DifferenceEntropy	***		glszm_LargeAreaLowGrayLevelEmphasis	NS *p* = 0.10
	glcm_DifferenceVariance	**		glszm_LowGrayLevelZoneEmphasis	NS *p* = 0.28
	glcm_Id	***		glszm_SizeZoneNonUniformity	NS *p* = 0.82
	glcm_Idm	***		glszm_SizeZoneNonUniformityNormalized	NS *p* = 0.05
	glcm_Idmn	***		glszm_SmallAreaEmphasis	**
	glcm_Idn	***		glszm_SmallAreaHighGrayLevelEmphasis	NS *p* = 0.58
	glcm_Imc1	NS *p* = 0.11		glszm_SmallAreaLowGrayLevelEmphasis	NS *p* = 0.35
	glcm_Imc2	*		glszm_ZoneEntropy	**
	glcm_InverseVariance	***		glszm_ZonePercentage	**
	glcm_JointAverage	*		glszm_ZoneVariance	NS *p* = 0.09
	glcm_JointEnergy	*	Neighbouring Gray Tone Difference Matrix	ngtdm_Busyness	***
	glcm_JointEntropy	*		ngtdm_Coarseness	***
	glcm_MCC	***		ngtdm_Complexity	*
	glcm_MaximumProbability	*		ngtdm_Contrast	**
	glcm_SumAverage	*		ngtdm_Strength	*
	glcm_SumEntropy	NS *p* = 0.85	IQ wavelets	IQwavelet_global	***
	glcm_SumSquares	NS *p* = 0.33		IQwavelet_local	***

## Data Availability

The data presented in this study and the python code used are openly available at https://github.com/AurelienCD/Resampling_Denoising_Deep_Learning_MRI (accessed on 21 December 2021).

## Supplementary Materials

The following are available online at https://www.mdpi.com/article/10.3390/cancers14010036/s1, Figure S1: Experimental paradigm and schema of the study. Figure S2: Bland–Altman plots showing the difference between predictive values obtained from radiomic model. Figure S3: Quantitative analyzes of the efficiency of the denoising DL model with the comparison with fast image concerning. Figure S4: ESPCNN and U-NET DL models comparison. Figure S5: Example of motion artefact corrected by the U-NET DL model. Figure S6: Pearson correlation of kurtosis, skweness, glcm MCC values between reference and fast and DL images. Table S1: Paired *t*-test of DL resampling impact on radiomic features. Table S2: Comparison of ICC for different radiomic models features between reference, fast image and DL images
