# Supplementary material for: The Impact of Resampling and Denoising Deep Learning Algorithms on Radiomics in Brain Metastases MRI
Source: Cancers (Basel). 2021 Dec 22;14(1):36. doi: 10.3390/cancers14010036 (PMC8750741; doi:10.3390/cancers14010036)
Supplement: Supplementary file 1 [file cancers-14-00036-s001.zip › cancers-1421173-supplementary.pdf]

Supp Table S1:

Classes	Features	Signicantly different
Intensity	Min	***
	Max	**
	Peak	NS p=0.06
	Mean	***
	Median	NS p=0.19
	Skewness	NS p=0.07
	Kurtosis	NS p=0.81
	CV(%)	**
	MaxOnMeanRing	**
	firstorder_10Percentile	***
	firstorder_90Percentile	***
	firstorder_Energy	NS p=0.35
	firstorder_Entropy	NS p=0.52
	firstorder_InterquartileRange	***
	firstorder_Kurtosis	NS p=0.82
	firstorder_Maximum	**
	firstorder_MeanAbsoluteDeviation	***
	firstorder_Mean	***
	firstorder_Median	***
	firstorder_Minimum	***
	firstorder_Range	NS p=0.62
	firstorder_RobustMeanAbsoluteDeviation	***
	firstorder_RootMeanSquared	***
	firstorder_Skewness	NS p=0.07
	firstorder_TotalEnergy	NS p=0.14
	firstorder_Uniformity	NS p=0.95
	firstorder_Variance	*
Gray Level Co-occurrence Matrix	glcm_Autocorrelation	**
	glcm_ClusterProminence	*
	glcm_ClusterShade	NS p=0.22
	glcm_ClusterTendency	*
	glcm_Contrast	**
	glcm_Correlation	*
	glcm_DifferenceAverage	**
	glcm_DifferenceEntropy	**
	glcm_DifferenceVariance	**
	glcm_Id	NS p=0.09
	glcm_Idm	NS p=0.25
	glcm_Idmn	**
	glcm_Idn	**
	glcm_Imc1	NS p=0.44
	glcm_Imc2	NS p=0.10
	glcm_InverseVariance	NS p=0.08
	glcm_JointAverage	**
	glcm_JointEnergy	NS p=0.98
	glcm_JointEntropy	NS p=0.24
	glcm_MCC	NS p=0.92
	glcm_MaximumProbability	NS p=0.49
	glcm_SumAverage	**
	glcm_SumEntropy	NS p=0.09
	glcm_SumSquares	*

Classes	Features	Signicantly different
Gray Level Dependence Matrix	gldm_DependenceEntropy	NS p=0.34
	gldm_DependenceNonUniformity	NS p=0.58
	gldm_DependenceNonUniformityNormalized	NS p=0.94
	gldm_DependenceVariance	NS p=0.65
	gldm_GrayLevelNonUniformity	NS p=0.69
	gldm_GrayLevelVariance	NS p=0.11
	gldm_HighGrayLevelEmphasis	*
	gldm_LargeDependenceEmphasis	NS p=0.88
	gldm_LargeDependenceHighGrayLevelEmphasis	**
	gldm_LargeDependenceLowGrayLevelEmphasis	*
	gldm_LowGrayLevelEmphasis	NS p=0.05
	gldm_SmallDependenceEmphasis	NS p=0.85
	gldm_SmallDependenceHighGrayLevelEmphasis	NS p=0.18
	gldm_SmallDependenceLowGrayLevelEmphasis	NS p=0.97
Gray Level Run Length Matrix	glrlm_GrayLevelNonUniformity	NS p=0.86
	glrlm_GrayLevelNonUniformityNormalized	NS p=0.96
	glrlm_GrayLevelVariance	NS p=0.11
	glrlm_HighGrayLevelRunEmphasis	*
	glrlm_LongRunEmphasis	NS p=0.85
	glrlm_LongRunHighGrayLevelEmphasis	*
	glrlm_LongRunLowGrayLevelEmphasis	*
	glrlm_LowGrayLevelRunEmphasis	NS p=0.06
	glrlm_RunEntropy	NS p=0.48
	glrlm_RunLengthNonUniformity	NS p=0.88
	glrlm_RunLengthNonUniformityNormalized	NS p=0.95
	glrlm_RunPercentage	NS p=0.95
	glrlm_RunVariance	NS p=0.76
	glrlm_ShortRunEmphasis	NS p=0.99
	glrlm_ShortRunHighGrayLevelEmphasis	*
	glrlm_ShortRunLowGrayLevelEmphasis	NS p=0.07
Gray Level Size Zone Matrix	glszm_GrayLevelNonUniformity	NS p=0.97
	glszm_GrayLevelNonUniformityNormalized	NS p=0.97
	glszm_GrayLevelVariance	NS p=0.28
	glszm_HighGrayLevelZoneEmphasis	NS p=0.06
	glszm_LargeAreaEmphasis	NS p=0.33
	glszm_LargeAreaHighGrayLevelEmphasis	NS p=0.99
	glszm_LargeAreaLowGrayLevelEmphasis	NS p=0.27
	glszm_LowGrayLevelZoneEmphasis	NS p=0.52
	glszm_SizeZoneNonUniformity	NS p=0.42
	glszm_SizeZoneNonUniformityNormalized	NS p=0.92
	glszm_SmallAreaEmphasis	NS p=0.82
	glszm_SmallAreaHighGrayLevelEmphasis	NS p=0.17
	glszm_SmallAreaLowGrayLevelEmphasis	NS p=0.64
	glszm_ZoneEntropy	NS p=0.73
	glszm_ZonePercentage	NS p=0.76
	glszm_ZoneVariance	NS p=0.33
Neighbouring Gray Tone Difference Matrix	ngtdm_Busyness	*
	ngtdm_Coarseness	*
	ngtdm_Complexity	NS p=0.14
	ngtdm_Contrast	*
	ngtdm_Strength	NS p=0.79
IQ wavelets	IQwavelet_global	***
	IQwavelet_local	NS p=0.50

Supp FIGURE S1:

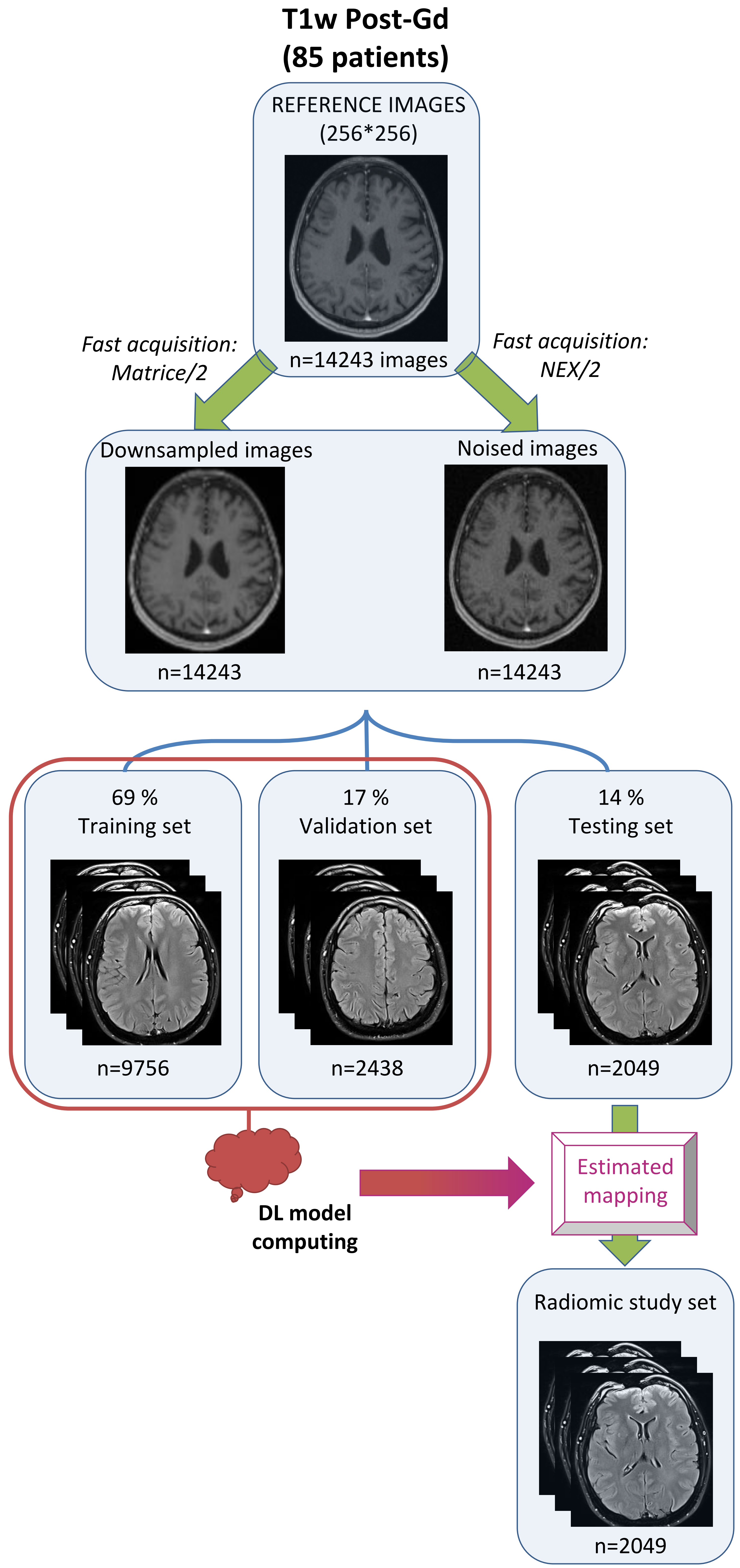

Experimental paradigm and schema of the study

Supp FIGURE S2:

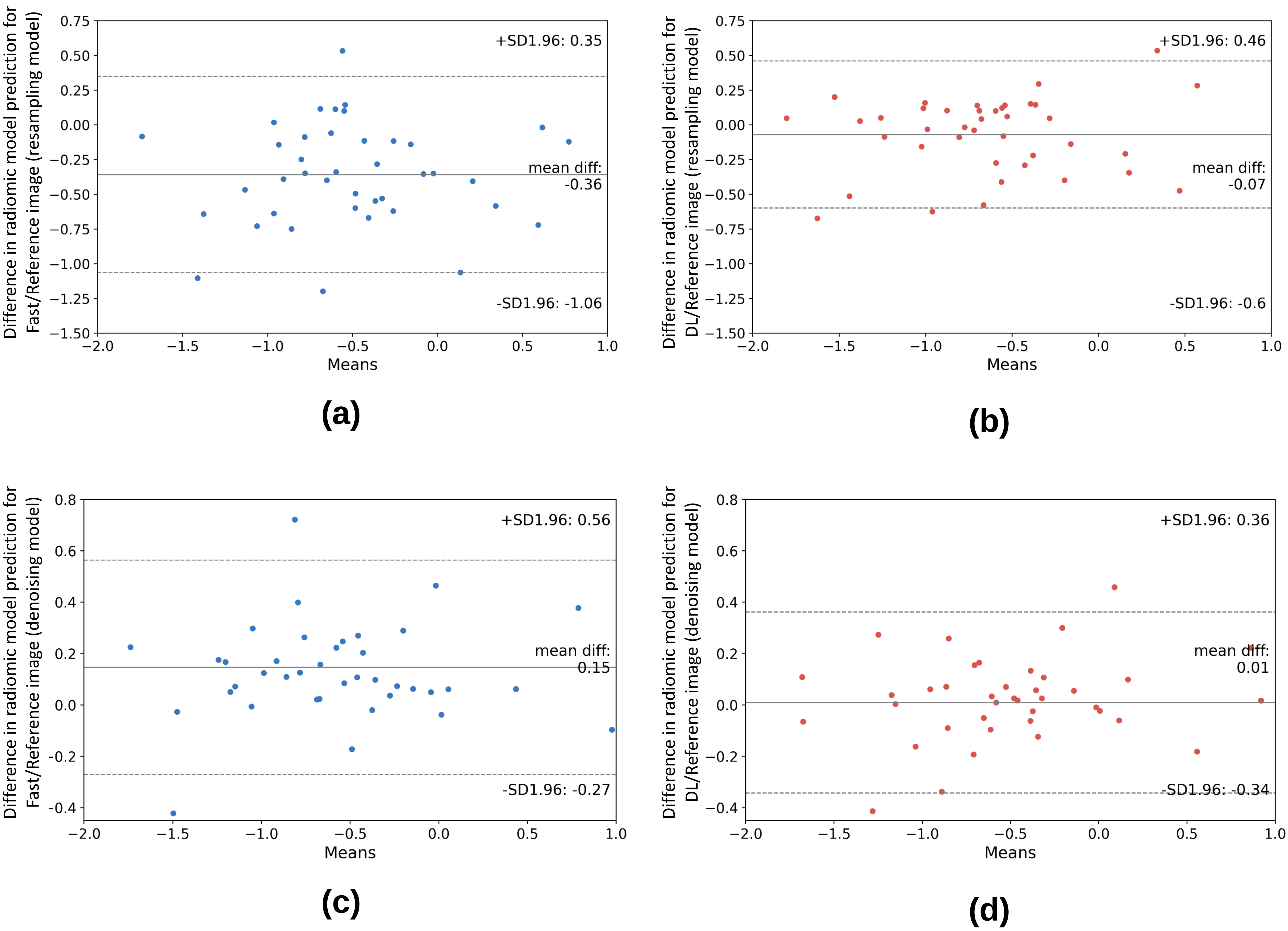

Bland–Altman plots showing the difference between predictive values obtained from radiomic model ([30]) from reference image to fast downsampling image (a) and DL resampling image (b) and reference image to fast noising image (c) and DL denoising image (d).

Supp FIGURE S3:

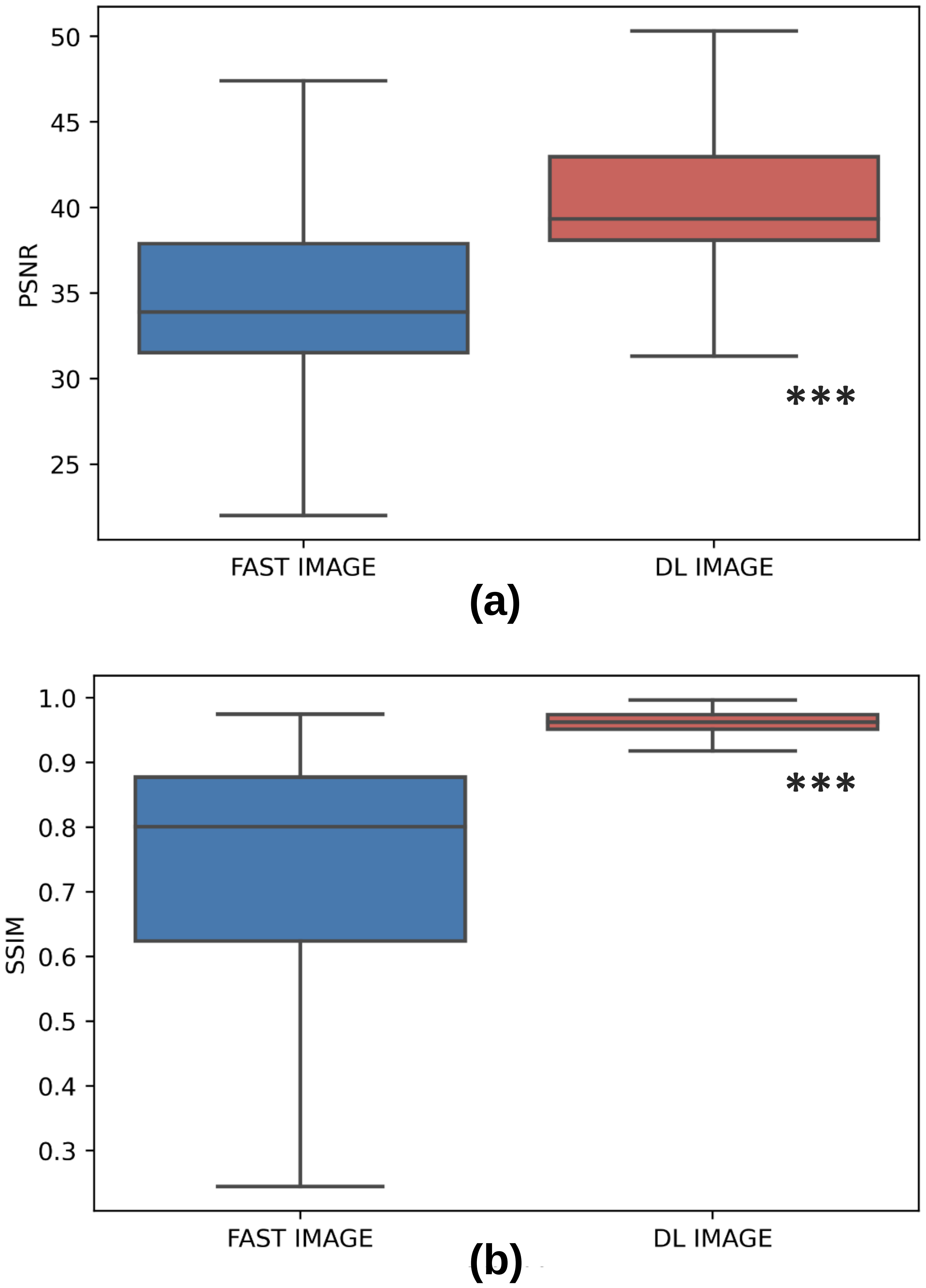

Quantitative analyzes of the efficiency of the denoising DL model with the comparison with fast image concerning PSNR (**a**) and SSIM (**b**) metrics. n=2049 for both groups, ***$p<0.001$ *vs* fast image.

Supp FIGURE S4:

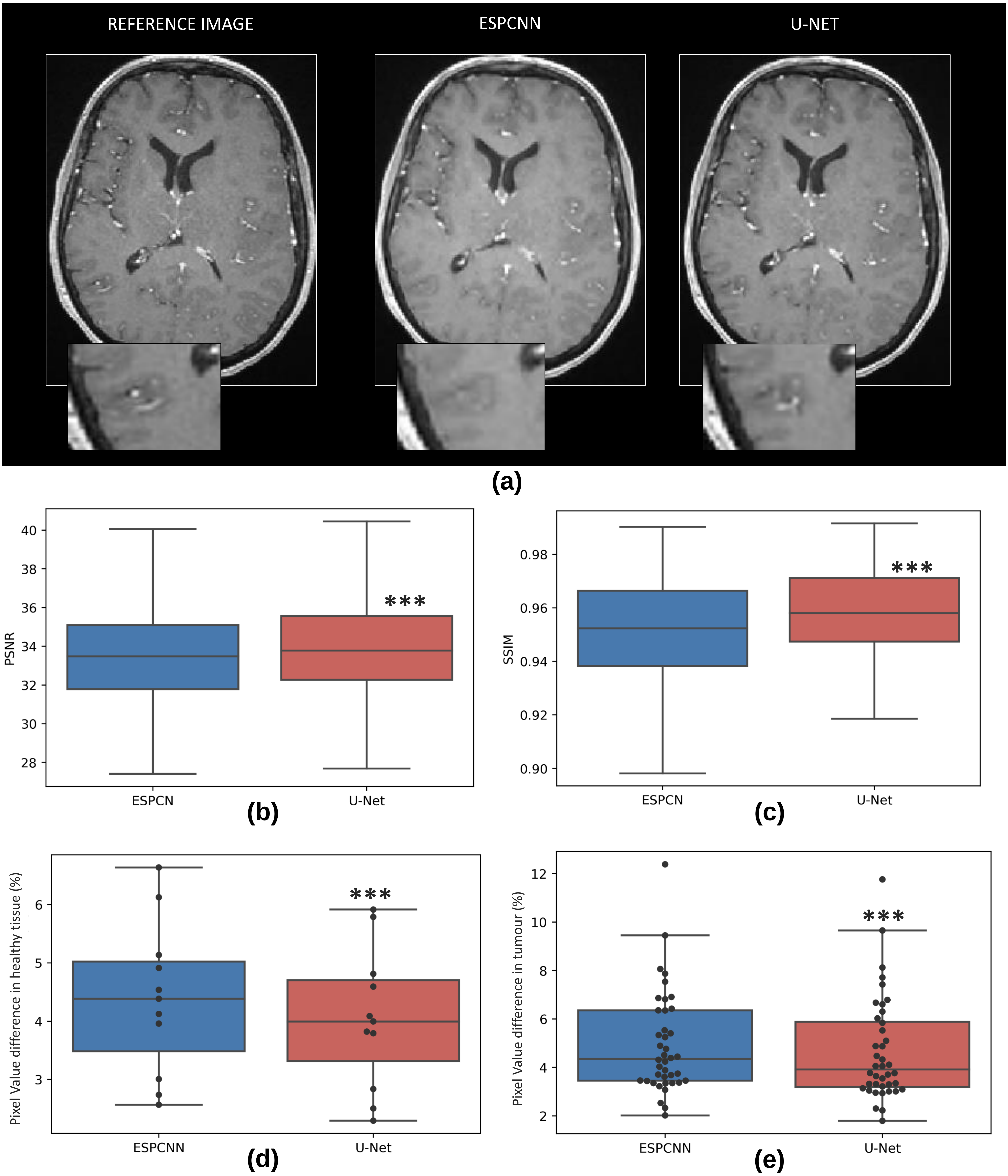

ESPCNN and U-NET DL models comparison. Representative MRI of reference images, ESPCNN reconstruction images and U-NET reconstruction images in healthy brain (**a**). Quantitative analyzes of the efficiency of the resampling ESPCNN and U-NET models concerning PSNR (**b**) and SSIM (**c**) metrics and concerning the pixel values difference with the original image in healthy tissue (**d**) and brain metastases (**e**). n=2049 for both groups, ***p<0.001 *vs* fast acquired image.

Supp FIGURE S5:

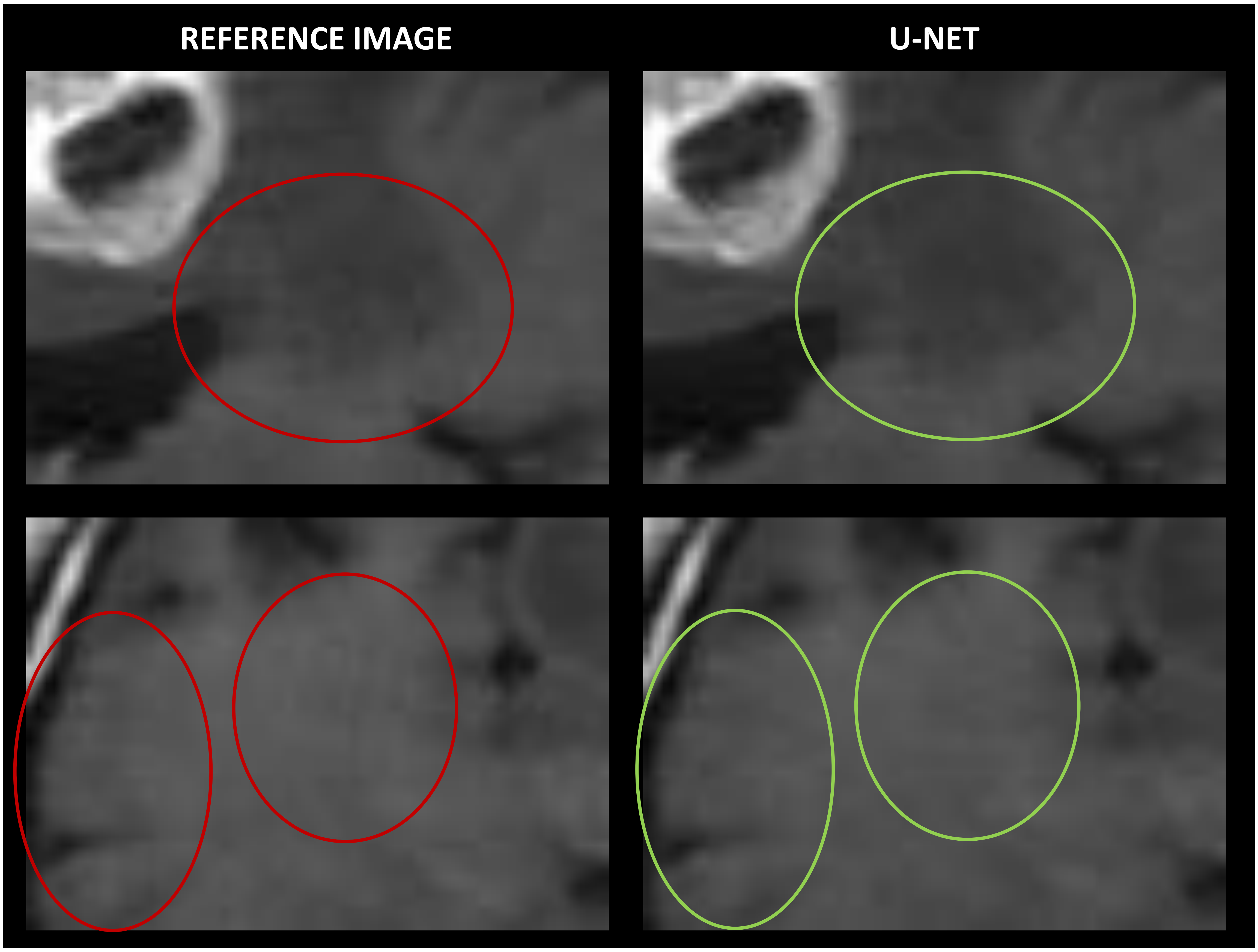

Example of motion artefact (red circles) corrected by the U-NET DL model (green circles).

Supp FIGURE S6:

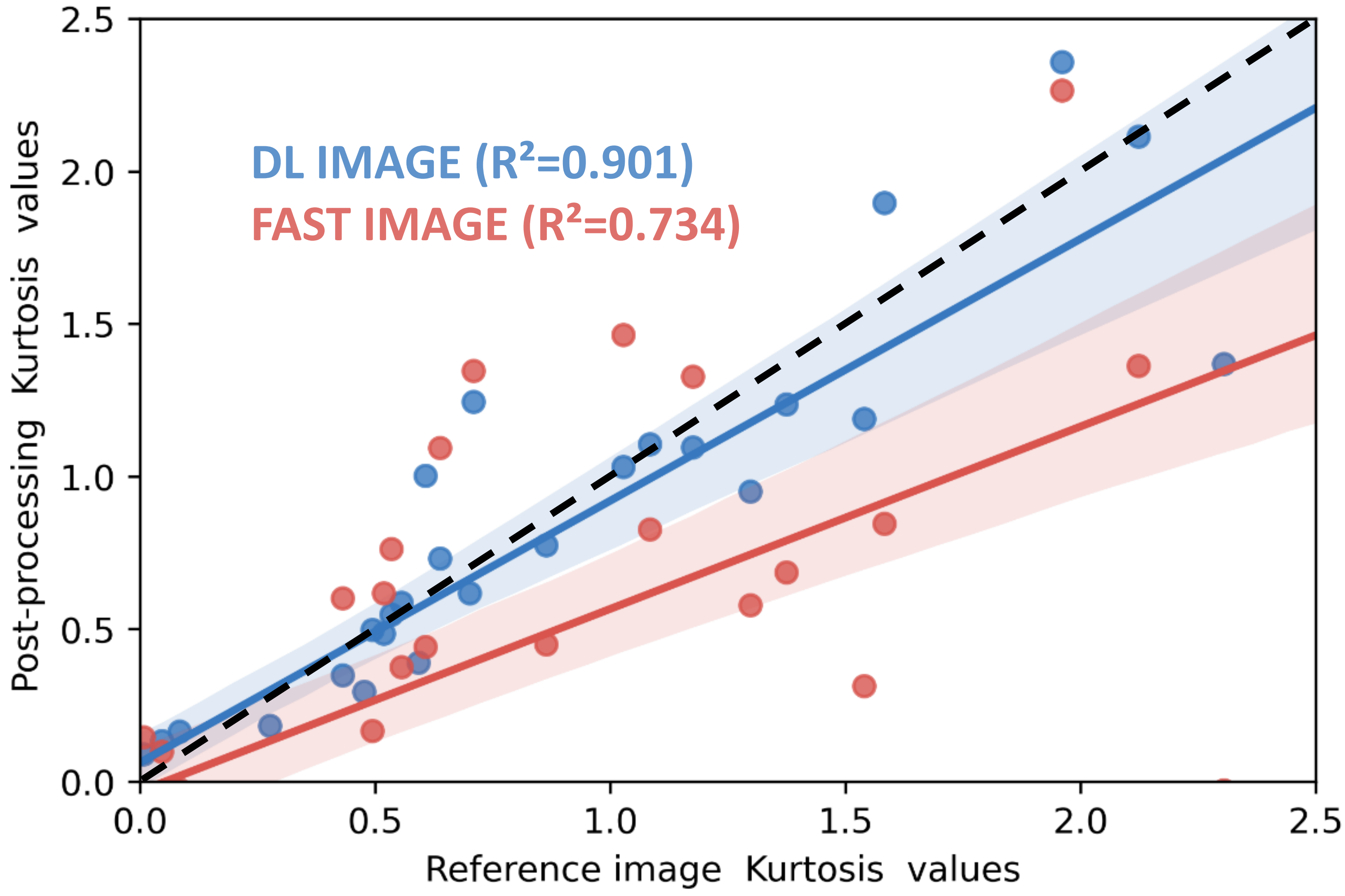

(a)

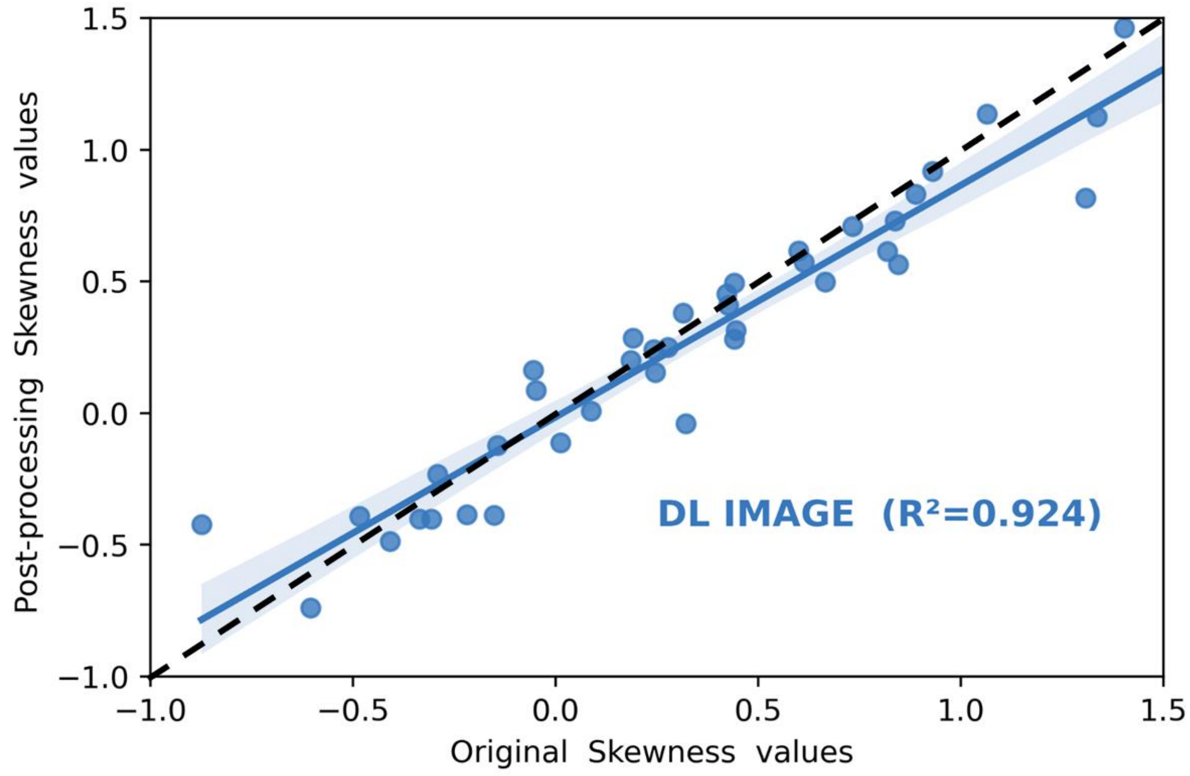

(b)

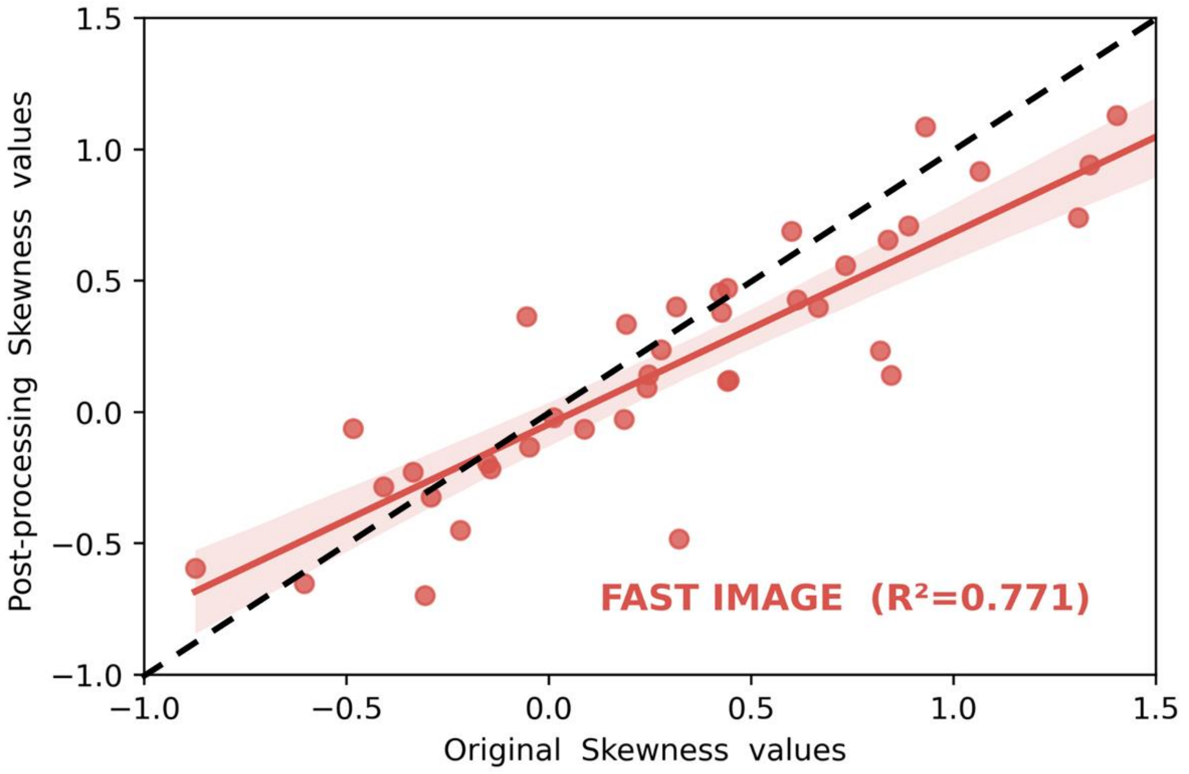

(c)

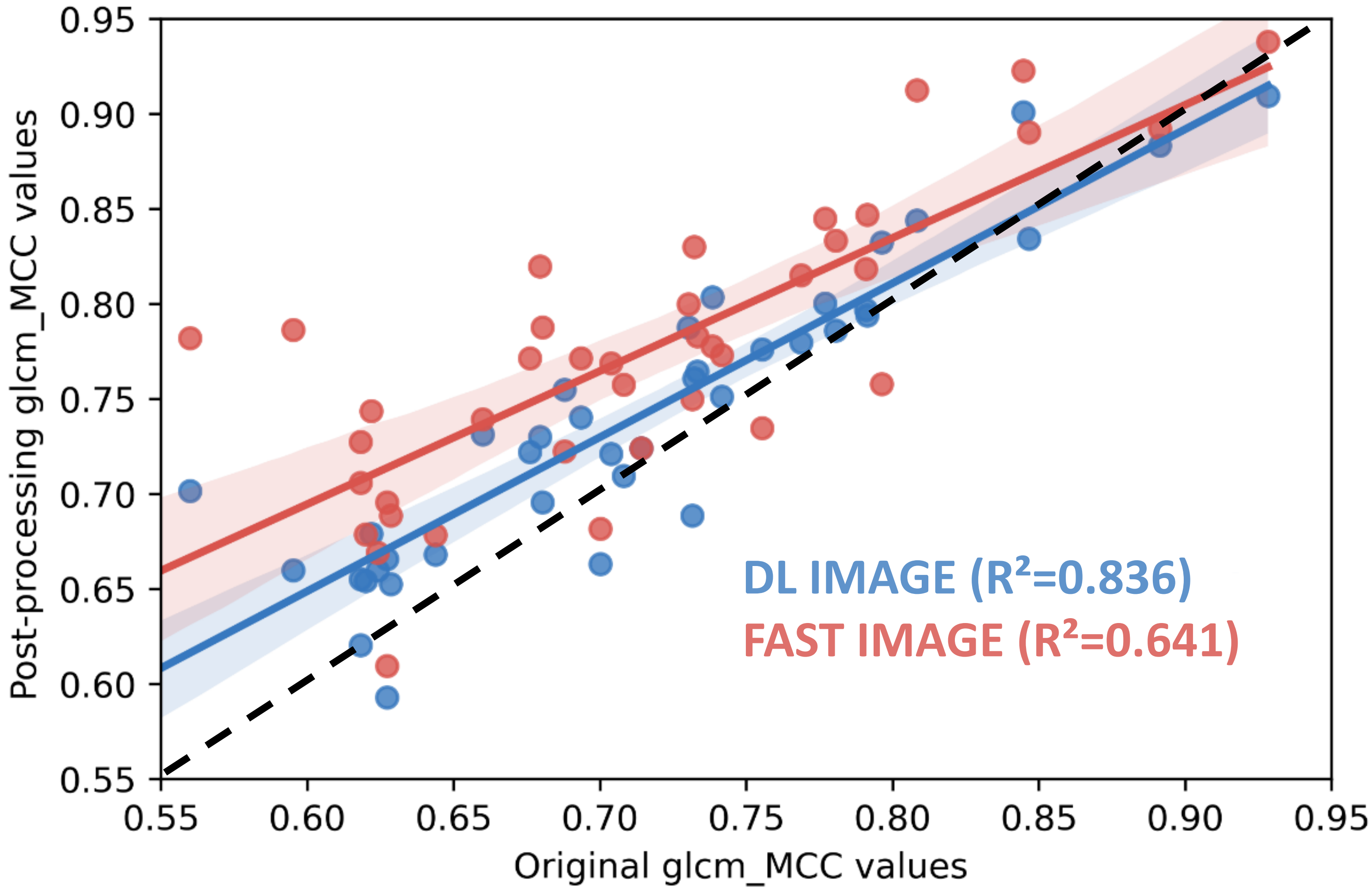

(d)

(**a**) Pearson correlation of kurtosis values between reference and fast and DL images. Pearson correlation of skweness values between reference and fast image (**b**) and DL images (**c**). (**d**) Pearson correlation of glcm_MCC values between reference and fast and DL images.

Supp Table S2:

Pulication	purpose	MRI	Accuracy		Software feature extraction	IBSI	Texture Selected features	Corresponding features with pyradiomics	Resampling		Denoised	
									Fast image	DL image	Fast image	DL image
Chien-Yi Liao et al. (2021)	Prediction treatment responce Local Tumor Control of NSCLC BM by GKRS	T1	Radiomics alone AUC=0,86	Radiomics combined with clinics AUC=0,95	MRP	Yes	GLCM homogeneity_LLH	firstOrder_uniformity				
		T1					GLCM correlation_LHL	glcm_correlation				
		T1					GLCM correlation_HLL	glcm_correlation				
		T1					GLCM correlation_HHL	glcm_correlation				
		T1 CE					GLCM correlation	glcm_correlation				
Chien-Yi Liao et al. (2021)	Prediction treatment responce Overal Survival of NSCLC BM by GKRS	T1	Radiomics alone AUC =0,64	Radiomics combined with clinics AUC=0,82	MRP	Yes	Maximum_LLL	Maximum				
		T1					Minimum_LLH	Minimum				
		T1					GLCM cluster tendency_LHH	glcm_cluster tendency				
		T1					GLCM correlation_HLL	glcm_correlation				
C-Q Su et al (2021)	Differentiation of solitary BM from GBM	T1-CE	AUC training 0,82	AUC validation0,81	AK software (Artificial Intelligence Kit, version 3.2.0, GE Healthcare, Shanghai, China).	No	RunLengthNonuniformity offset4 SD	RunLengthNonuniformityNormalised				
							MeanDeviation	MeanAbsoluteDevation				
							GLCMEntropy_Offset1_SD	GLCM_DifferenceEntropy				
							Volume CC	NA	NA	NA	NA	NA
							GLCMEntropy_offset4_SD	GLCM_SumEntropy				
							GLCMEnergy_offset7_SD	GLCM_JointEnergy				
							ClusterProminence_offset7_SD	GLCM_ClusterProminece				
							GLCMEnergy_angle135_offset7	GLCM_JointEnergy				
							LongRunHighGreyLevelAmphasis	LongRunHighGreyLevelAmphasis				
							kurtosis	kurtosis				
							GLCMEntropy_angle45_offset4	GLCM_JointEntropy				
Qian et al. (2019)	Differentiation of BM from GBM	T1-CE	5 fold Cross validation AUC 0,9[0,74-0,96]		PyRadiomics	Yes	Maximum	Maximum				
							Skewness	Skewness				
							Kurtosis	Kurtosis				
							Minimum	Minimum				
							10Percentile	10Percentile				
							Kurtosis	fisrtorder_Kurtosis				
							Flatness	NA	NA	NA	NA	NA
							Maximum2DDiameterSlice	NA	NA	NA	NA	NA
							Sphericity	NA	NA	NA	NA	NA
							Correlation	glcm_Correlation				
							InverseDifferenceMomentNormalized	glcm_IDifferenceMomentNormalized				
							InverseDifferenceNormalized	glcm_InverseDifferenceNormalized				
							Informational Measure of Correlation 1	glcm_Informational Measure of Correlation 1				
							Informational Measure of Correlation 2	glcm_Informational Measure of Correlation 2				
							InverseDifferenceNormalized	glcm_InverseDifferenceNormalized				
							JointEntropy	JointEntropy				
							InverseDifferenceMomentNormalized	glcm_IDifferenceMomentNormalized				
							Informational Measure of Correlation 1	glcm_MCC				
							ShortRunLowGrayLevelEmphasis	ShortRunLowGrayLevelEmphasis				
							LargeAreaLowGrayLevelEmphasis	glszm_LargeAreaLowGrayLevelEmphasis				
							ZoneEntropy	glszm_ZoneEntropy				
							LargeAreaHighGrayLevelEmphasis	glszm_LargeAreaHighGrayLevelEmphasis				
							SizeZoneNonUniformity	SizeZoneNonUniformity				
							ZoneEntropy	ZoneEntropy				

Comparison of ICC for different radiomic models features between reference, fast image and DL images. Red color correspond to an ICC < 0.85 and green to an ICC > 0.85.
